# The rise of hierarchy and modularity in biological networks explained by Empedocles’ double tale ∼2,400 years before Darwin and systems biology

**DOI:** 10.3389/fgene.2022.973233

**Published:** 2022-08-17

**Authors:** Gustavo Caetano-Anollés, Richard Janko

**Affiliations:** ^1^ Evolutionary Bioinformatics Laboratory, Department of Crop Sciences, C.R. Woese Institute for Genomic Biology, and Illinois Informatics Institute, University of Illinois, Urbana, IL, United States; ^2^ Department of Classical Studies, University of Michigan, Ann Arbor, MI, United States

**Keywords:** biphasic bow-tie pattern, empedocles, hourglass, molecular structure, evolutionary diversification, evolutionary growth, phylogenetic analysis, papyrus

## Introduction

Networks describe how parts interact with each other and associate to form integrated systems. These interactions can be modeled with graphs. The vertices (*nodes*) of a network describe parts, while lines (*links*) that connect vertices describe pairwise interaction between them. Value functions are often mapped onto the nodes and links of the networks to describe static or dynamic phenomena. Networks are often structured by modularity and hierarchy across timescales (Bogdan et al., 2021). Modules make up communities, typically sets of nodes that are more connected with each other than with other nodes of the network (though link communities also exist and can be dissected; Ahn et al., 2010). Hierarchy embodies an organization that is ranked to some authority, with parent-child relationships influenced by levels, nesting, balance and authorities of the system, i.e., “*a system that is composed of interrelated subsystems*, *each of the latter being in turn*, *hierarchic in structure until we reach some lowest level of elementary subsystem*” (Simon, 1962). In the context of networks, hierarchical modularity is simply the fractal-like reuse or embedding of simpler network modules into modules of higher complexity.

**FIGURE 1:**
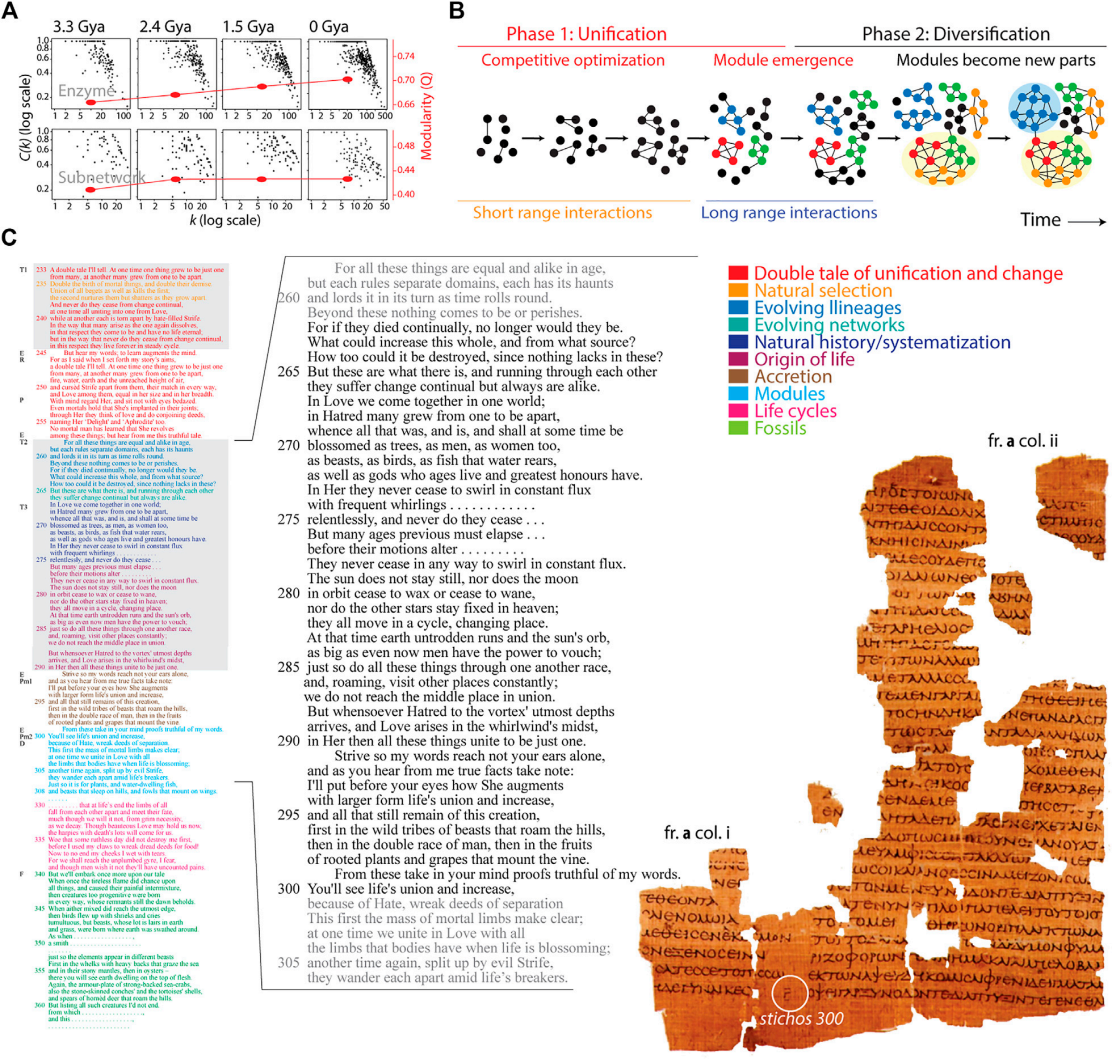
The evolutionary structuring of biological networks explained by the poems of *P. Strasb. Gr.* Inv. 1665–6, a ∼2,000 year-old papyrus found in the ancient city of Panopolis in Upper Egypt. **(A)**. Log-log plots of the clustering coefficient *C* (*k*) as a function of the number of links *k* for enzyme and subnetwork one-mode projections of an evolving bipartite network of the enzymes and subnetworks of metabolism. The networks describe how metabolism grows in evolutionary time, with time unfolding in billions of years (Gy) according to a clock of protein folds. The scaling is the hallmark of hierarchical modularity, it increases in evolution, and is stronger at lower levels of metabolic organization. Modularity (*Q*) measures connectivity density in node communities and increases in metabolic network evolution. Data from Mughal and Caetano-Anollés (2019). **(B)**. A biphasic model of module creation illustrates the emergence of hierarchical modularity in evolution of networks. Nodes of the network are parts of a growing system and links describe their interactions. The larger the number of links the more cohesive is the structure of a subnetwork. The rise of hierarchical modularity in Phase 1 results in small highly connected subnetworks, which give rise to modules. In Phase 2, these emergent modules become new parts, which coalesce by combination into higher modules (highlighted with shades). **(C)**. Indexed translation of *P. Strasb. Gr.* Inv. 1665-6 (left) with segments colored according to themes (see Supplementary Material for thematic analysis). The translation of ensemble fragment (fr.) **a** of the ancient papyrus, with its two columns (right), reconstructs lines 262–300 of *Empedocles’ On Nature*. Note the scribal stichometric sign Γ, which indicates that the line corresponds to v. 300 of *Physika* Book I quoted by Simplicius. Text lines of theses 2 and 3 (T2 and T3) of the translation are indexed with numbers and are highlighted in black when they are part of ensemble fr. **a**. Please refer to Janko (2004) for the original Ionic text of the translation.

Hierarchy and modularity are pervasive in biological networks and arise naturally as long as there is an underlying cost of emerging links (Ravasz et al., 2002; Clune et al., 2013; Corominas-Murtra et al., 2013; Mengitsu et al., 2016). We have explained the rise of hierarchical modularity in networks with a biphasic (*bow-tie*) theory of module emergence (Mittenthal et al., 2012), which relates to things that grow (Caetano-Anollés et al., 2018). The theory is compatible with modeling frameworks that reveal hierarchical modularity induces an “hourglass” effect in which networks channel many inputs to produce many outputs through a core of intermediate nodes (Sabrin and Dovrolis, 2017). We used chronologies to test the rise of hierarchical modularity in evolutionary time (Caetano-Anollés et al., 2019). Chronologies arrange parts or interactions in the order of their temporal or irreversible occurrence. They have been reconstructed using phylogenomic methods from genomic data from thousands of organisms and viruses (Caetano-Anollés et al., 2021). Chronology-driven time series of networks (*evolving networks*) uncovered the emergence of hierarchical modularity in networks at different time scales, including the nanosecond-dynamics of proteins, the rewiring of metabolomic and transcriptome-informed metabolic networks, and deep-time evolving networks describing the evolution of metabolism, an “elementary functionome” of functional protein loops, and protein domain organization (e.g., Aziz et al., 2016; Mughal and Caetano-Anollés, 2019; Aziz and Caetano-Anollés, 2021). For example, an evolving bipartite network of metabolism that links enzymes to subnetworks of the KEGG metabolic pathway database can be dissected into its two one-mode network projections, both of which increase hierarchy and modularity as they evolve along a timeline of billions of years of evolution (Figure 1A). Constraints on network structure were however stronger at the enzyme level suggesting a “principle of granularity” that confirms Simon’s prediction that lower organizational levels should exhibit stronger internal links (Simon, 1962).

Here we discuss how our model of unification and diversification has been already described in a ∼2,000-year-old papyrus from the ancient city of Panopolis in Upper Egypt. The embedded poem, which is attributed to Empedocles of Akragas [Ἐμπεδοκλῆς (*Empedoklēs*); ca. 495-435 BC], recounts a “double tale” of unification and change that is consistent with the biphasic theory of module emergence. We interpret Empedocles’ ancient text as a description of biological evolution with network hierarchies ∼2,400 years before Darwin and systems biology.

## A phylogenomic-based biphasic model of module generation is a double tale of growth

The biphasic theory of module emergence explains evolutionary growth, a process known as *accretion* (Mittenthal et al., 2012). In a first phase, parts are at first weakly linked and associate variously. As they diversify, they compete with each other and are often selected for performance. The emerging interactions constrain their structure and associations. This causes parts to self-organize into modules with tight linkage. In a second phase, variants of the modules diversify and become new parts for a new generative cycle of higher-level organization. Figure 1B illustrates how competitive optimization of parts trigger the emergence of network communities (modules) in a dynamic process of system innovation, and how this emerging modular structure diversifies and generates new parts for a combinatorial landscape of increasing organization. The paradigm is a “double tale” that predicts the rise of hierarchical modularity in evolving networks. This prediction has been experimentally confirmed at different timescales and complexity levels.

## Empedocles’ *On Nature*, *P. Strasb. Gr.* Inv. 1665-6

In 1904, German archaeologist Otto Rubensohn purchased a late first century AD roll for *Das Papyruskartell* from an antiquities shop in Akhmim, Egypt. The roll was part of a collar-shaped funeral pectoral wreath that was originally attached to a mummy recovered from a nearby necropolis of the ancient city of Panopolis. The 52 papyrus fragments contained text written in columns of 30 hexameters each. They were conserved at the National University Library of Strasbourg in 1905 but were not transcribed or translated until papyrologist Alain Martin attributed the text in them to Empedocles in 1992. Martin, together with Oliver Primavesi, published a textual reconstruction, transcription, paleographic commentary and interpretations in *L’Empédocle de Strasbourg* (the *editio princeps*) 7 years later (Martin and Primavesi, 1999).

The discovery of the Strasbourg papyrus (*P. Strasb. Gr.* Inv. 1665-6) is of extraordinary significance. Very much like the carbonized Derveni papyrus from Macedonia (Kouremenos et al., 2006; Kotwick, 2017), it opened a floodgate of reinterpretations of Presocratic philosophy (Mace, 2002; Janko, 2004; Trépanier, 2017, 2019; Vassallo, 2019). The stichometric symbol Γ, third of the 24 letters of the Ionic alphabet that scribes placed in the left-hand margins of their texts (Figure 1C), denotes the 300th line of verse (Van der Ben, 1999). It shows that the text is a copy *via* scribal transmission of at least 300 lines of a comprehensive philosophical treatise. Textual reconstructions matched doxographic evidence, and in particular a long passage of Empedocles’ poem *On Nature* quoted by Simplicius (a Neoplatonist commentator on Aristotle) who often quoted from Theophrastus (Mansfeld, 2016). These overlaps settled some disagreements about quotations in Diogenes Laërtius and the *Suda* and clarified Empedocles’ account of his dynamic model of nature (known as “cosmic cycle”), the interpretation of which had been controversial (Graham, 1988; Trépanier, 2000). Textual reconstructions also demanded a revision of traditional interpretations derived mostly from Aristotle and his commentators, who disparaged Empedocles because they embraced a static Universe. The fragments of the Strasbourg papyrus remained disjointed and their assembly peculiar; a reinterpretation permitted the reconstruction of a largely uninterrupted passage of coherent philosophical poetry that is beautiful, novel and puzzling (Janko, 2004, 2010; Trepanier, 2019). This passage suggested that Empedocles’ poems, *On Nature*, which dealt with the creation of the living world, and *Purifications*, which focused on the fate of the soul, presented a unified theory that described the physical nature of living matter, and consequently, was a poetical rendition of a coherent philosophy.

### Empedocles’ double tale describes evolution of biological networks

Empedocles’ *On Nature* embodies a “double tale” of evolutionary growth and change in which two opposing forces unify and diversify. Zirkle (1941) intimated almost a century ago that Empedocles’ theory described biological evolution ∼2,400 years before Darwin—a claim that was based at that time on quotations from Aristotle and Lucretius. Our recent biological reinterpretation of *On Nature* based on *P. Strasb. Gr.* Inv. 1665-6 supports this contention (Caetano-Anollés and Janko, 2021). Remarkably, the double tale also involves a “network” paradigm (*tela vitae*) of systems of interconnected things. Figure 1C presents a thematic indexing of the poem, highlighting segments describing the double tale, natural selection, evolving lineages, evolving networks, natural history and systematization, origin of life, accretion, modules, life cycles, and fossil remnants. An indexed translation and commentary can be found in Supplementary Material and in Caetano-Anollés and Janko (2021).

The first three lines of the poem (lines 233–235, = Diels-Kranz (DK) fr. no. B 17.1-3) introduce the main thesis of Empedocles’ argument:

“*A double tale I’ll tell. At one time one thing grew to be just one*
*From many, at another many grew from one to be apart*.
*Double the birth of mortal things, and double their demise.*”

This thesis describes the unification and diversification of things that are “*mortal*” (θνητóς) and “*grow*”. One process grows these living things by “*union*” (Love, Φιλóτης), while the other grows things “*apart*” into many distinct forms (Strife, Νεῖκος). Since growing apart implies that unified things become separated by a distance in time and space, one very likely interpretation of his crucial statement about growth is that it describes a process of evolutionary diversification. Note that there is no evidence in the text that “things” that unify or diversify should refer exclusively to Empedocles’ “elements” (fire, water, earth and air, listed in line 249), as has been claimed by encyclopedic editions or other interpretations that give great weight to Roman doxographic evidence (e.g. Trépanier, 2017). In fact, line 235, “*Double the birth of mortal things, and double their demise*”, crucially reinforces the biological rather than the “cosmic cycle” interpretation. A balance of birth and demise in biology implies natural selection and change, the hallmarks of Darwinian evolution*.* Natural selection requires gains through birth of reproducing entities that “*double*” as they grow. Demise counterbalances growth through either stasis (unproductive growth) or death. Implicit in this process (or other causal influences) is differential loss and reproduction as prelude to fitness.

The lines that follow restate the main thesis but now describe the frustrated dynamics of the two tales (lines 236–240, = DK fr. no. B 17.4–9), anticipating the persistent and ephemeral properties of evolving systems (lines 241–244, = DK fr. no. B 17.10–13). Subsequent text reinforce the main thesis step by step, *via* exhortation and the gradual revelation of Empedocles’ argument (Janko, 2010), which now anticipates concepts in systems biology. For example, the text corresponding to the papyrus fragment ensemble fr. **a** (illustrated in Figure 1C) presents crucial principles that are common to modern evolutionary biology, sometimes cryptically evident (Caetano-Anollés and Janko, 2021). Lines 258–260 specify how growing things establish a *hierarchy* of wholes unified from integrated parts to make up what can be interpreted as lineages: “*For all these things are equal and alike in age but each rules separate domains*”. Indeed, the rise of lineages from a ‘last universal common ancestor’ endows them with equal age, a property that enables the Sibley-Ahlquist model used for calculation of stem and crown ages of higher taxa (Stadler et al., 2014). Lines 261–266 crucially extend the concept of lineages of a hierarchy (a tree) to lineages of an evolving network: “*But these are what there is*, *and running through each other they suffer change continual but always are alike*”. Lines 270–274 posit that lineages of the network “*blossom*” into species, “*as trees*, *as men*, *as women too*, *as beasts*, *as birds*, *as fish that waters rear*”. Lines 285–287 later restate how parts “*through one another race, and, roaming, visit other places constantly*” as they unify in the context of an origin of life, making explicit time trajectories of evolutionary recruitment that make network structures. Finally, lines 293–298 describe how unification “*augments with larger form life*’*s union and increase*” to generate a wealth of organismal diversity, which is then catalogued. Remarkably, these interpretations of recurrent arguments gradually advance two concepts linked to the double tale, growth and diversification, which are recurrent features in evolutionary and systems biology research and central elements of our biphasic model.

## Conclusion

Empedocles’ double tale of evolutionary growth represents a discovery of extraordinary significance. It is one of few Presocratic texts preserved by direct scribal transmission. The double tale coherently explains the living world with a network paradigm of accretion and change. This ancient philosophy embodies a biphasic model of module generation in biological systems, which explains fractal-like patterns of complexification that are both entrenched and highly dynamic at all levels of organization. The themes that are advanced in the papyrus have considerable explanatory power, given background knowledge and evidence from evolutionary genomics and systems biology. This fact in itself now demands explanation.
