# Erratum: The rise of hierarchy and modularity in biological networks explained by Empedocles’ double tale ∼2,400 years before Darwin and systems biology

**DOI:** 10.3389/fgene.2022.1036961

**Published:** 2022-09-27

**Authors:** Frontiers Production Office

**Affiliations:** Frontiers Media SA, Lausanne, Switzerland

**Keywords:** biphasic bow-tie pattern, empedocles, hourglass, molecular structure, evolutionary diversification, evolutionary growth, phylogenetic analysis, papyrus

Due to a production error, the names of Empedocles and Darwin in the article title were erroneously written in lowercase.

The publisher apologizes for this mistake. The original article has been updated.

