# Supplementary material for: The rise of hierarchy and modularity in biological networks explained by Empedocles’ double tale ∼2,400 years before Darwin and systems biology
Source: Front Genet. 2022 Aug 17;13:973233. doi: 10.3389/fgene.2022.973233 (PMC9428273; doi:10.3389/fgene.2022.973233)
Supplement: Supplementary file 1 [file DataSheet1.PDF]

Supplementary Material

Thematic indexing and interpretation of Empedocles' *On Nature I, P. Strasb. Gr. Inv.* 1665-6

Lines are indexed with numbers and also arguments: T, main thesis; E, exhortation; R, reprise; P, proof; Pm, promise; D, digression; and F, fulfillment of promise. The three theses of the papyrus provide the grounds for fundamental philosophy. Please refer to Janko (2004) for the original Greek text of the translation. Text is colored according to central themes, which are briefly interpreted in text boxes. More extensive elaborations can be found in Caetano-Anollés and Janko (2021). Note how themes are integrated into the narrative in careful succession (see footnote below).

A double tale I'll tell. At one time one thing grew to be just one T1
from many, at another many grew from one to be apart.
Double the birth of mortal things, and double their demise.
Union of all begets as well as kills the first;
the second nurtures them but shatters as they grow apart.
And never do they cease from change continual,
at one time all uniting into one from Love,
while at another each is torn apart by hate-filled Strife.
In the way that many arise as the one again dissolves,
in that respect they come to be and have no life eternal;
but in the way that never do they cease from change continual,
in this respect they live forever in a steady cycle.
But hear my words; to learn augments the mind. E
For as I said when I set forth my story's aims, R
a double tale I'll tell. At one time one thing grew to be just one
from many, at another many grew from one to be apart,
fire, water, earth and the unreached height of air,
and cursèd Strife apart from them, their match in every way,
and Love among them, equal in her size and in her breadth.
With mind regard Her, and sit not with eyes bedazed. P
Even mortals hold that She's implanted in their joints;
through Her they think of love and do conjoining deeds,
naming Her 'Delight' and 'Aphrodite' too.
No mortal man has learned that She revolves
among these things; but hear from me this truthful tale. E

Double tale of unification and change:

The double tale embodies two opposing forces of evolutionary growth. One unifies (Love) and the other diversifies (Strife) in a landscape of continual change. The double tale represents the main thesis of the poem (T1). The repeated 'one from many' allegory can be interpreted in modern biology as processes of multiple origination leading to growth and cohesive structure, such as the generation of biological modules at different levels of organization, or processes of accretion, recruitment and horizontal exchange leading to the reuse of biological innovations across space and time. In contrast, the 'many from one' allegory can be interpreted as processes of evolutionary diversification attuned with those of heredity and population genetics. The text highlights the immutability of the succession of the two forces of biological growth, their recurrent struggle, the ephemeral nature of parts and wholes that are unified and diversified, and their continual growth. While nothing in the text prevents interpreting the opposing forces as acting concurrently, their combination in a 'double tale' suggest a crucial integration (at one time, at another), which is in line with the biphasic (bowtie or hourglass) theory of module emergence that explains the rise of hierarchy and modularity (community structure) in biological systems modelled with networks (e.g. proteins, cells, organisms and cities; Caetano-Anollés et al., 2018). The reprise (R, lines 246-251), shifts the context of the double tale from living beings to the elements (fire, water, earth and air) of an evolving planetary realm. This shift likely follows contemporary atomistic views (Leucippus) and/or ideas of energy-originating universal flows (Heraclitus) that would apply to life's chemical makeup. The proof (P, lines 252-256) shows how the unification force ('Her', 'She', 'Joy', 'Delight', 'Aphrodite') is 'implanted' in parts (joints) and wholes (mortals) of humans and is made vivid in sexual reproduction, which fosters genetic recombination processes (e.g. conjugation, meiosis) that unify parental genetic materials in the offspring or fosters processes of social unification of partners that benefit the rearing of the progeny.

Natural selection:

Evolutionary growth involves the birth and death of 'mortal things' in a struggle for unification and diversification of biological parts (limbs; lines 301-306) and wholes (organisms; lines 170-273) that is typical of evolutionary processes broadly defined: variation, innovation, struggle for existence, competition, natural selection, adaptation, optimization, and recurrent change and exchange (including those typical of Darwinian evolution). That 'birth' and 'demise' are part of a struggle for existence in a battle for resources is made vividly evident in lines 335-336 of the 'Life cycles' theme: *'Woe that some ruthless day did not destroy me first, before I used my claws to wreak dread deeds for food!'*.

T2 For all these things are equal and alike in age,
but each rules separate domains, each has its haunts
and lords it in its turn as time rolls round.
Beyond these nothing comes to be or perishes.
For if they died continually, no longer would they be.
What could increase this whole, and from what source?
How too could it be destroyed, since nothing lacks in these?
But these are what there is, and running through each other
they suffer change continual but always are alike.
T3 In Love we come together in one world;
in Hatred many grew from one to be apart,
whence all that was, and is, and shall at some time be
blossomed as trees, as men, as women too,
as beasts, as birds, as fish that water rears,
as well as gods who ages live and greatest honours have.
In Her they never cease to swirl in constant flux
with frequent whirlings . . . .
relentlessly, and never do they cease . . .

Evolving lineages:

The second and third theses of the poem (T2 and T3) apply the double tale to things that are ‘equal and alike in age’. These things are wholes that result from unification of parts (since T2 and T3 follows P, which focuses on Love). Because they are alike in age (they have a same temporal origin) and they are later referred to as ‘all that was, and is, and shall at some time be blossomed as [organisms]’ (lines 269-273), they must be interpreted as evolving lineages. These diversifying wholes are also considered ‘equal’, very much as lineages of a phylogeny at any moment in time become taxa, palpable entities of a same rank. The fact that wholes rule ‘separate domains’ of life as ‘time rolls round’ is expected in lineages as they travel from an origin, a last universal common ancestor (LUCA), to the present via different phylogenetic paths. This results in a hierarchy of taxonomic wholes, a tree-like structure, a ‘Tree of Life’. Finally, nothing but the unified wholes form and perish in the lineages, fulfilling a continuum of change. In this regard, if organisms ‘died continually’ they would break the continuity of lineages of organisms of those lineages would become extinct. The two questions posed reinforce the sufficiency of lineages of wholes as *explanandum* of diversity.

Evolving networks:

Lines 265-266 of T2 are remarkable. Lineages ‘running through each other’ turn the previously defined ‘Tree of Life’ into a ‘Network of Life’. This evolving network preserves both hierarchy and identity (lineages rule ‘separate domains’ and are always ‘alike’ despite suffering ‘change continual’). Thus, the poem accounts for horizontal processes that have been intimated central only recently in modern evolutionary biology, such as horizontal gene transfer, recruitment, and parallel evolution. It also accounts for the existence of networks that are structured, supporting the presence of both hierarchy and modularity in biological networks. Network thinking represents a fundamental conceptual change in organization. It was not revisited until the time of Linnaeus in the mid 1700s and was not embraced by evolutionary biologists until the beginning of this century. Note that lines 285-290 of the ‘Origin of life’ theme recap the concept of networks at a time when lineages were not present: *‘just so do all these things through one another race, and, roaming, visit other places constantly’*. This crucially makes the evolving networks not only relevant to organismal wholes but to other biological parts and wholes.

Natural history/systematization:

T3 extends the double tale to the living world of organisms. The abstract ‘things’ of T1 that are unified and diversified now become biological observables such as organisms (tangible to the philosopher: trees, fish, birds, beasts, men). We interpret T3 as a natural history description of our living world, i.e. an attempt to create a *Systema Naturae* of classification. However, this natural history is described as a global unification force that gives rise to a ‘world’ of evolving organisms. The ‘we’ and coming together in ‘one world’ has been controversial because of mismatches between doxography (Simplicius) and scribal transmission (van der Ben, 1999; Trépanier, 2017). The original translation uses ‘single ordered whole’ instead of ‘one world’. Perhaps ‘we’ personifies the double tale by illustrating the emergence of complex wholes (organisms) from parts induced by the unification force. The force (‘Love’, ‘Her’) is highly dynamic, surprisingly, being described in the papyrus by fluid spiraling motions (‘swirls’, ‘whirlings’, ‘dense eddies’ that are in ‘constant flux’)(van der Ben, 1999). Thus, the evolving networks of life are dynamic and their links fluid as fluxes ‘through one another race’.

But many ages previous must elapse . . .
before their motions alter . . . .
They never cease in any way to swirl in constant flux.
The sun does not stay still, nor does the moon
in orbit cease to wax or cease to wane,
nor do the other stars stay fixed in heaven;
they all move in a cycle, changing place.
At that time earth untrodden runs and the sun's orb,
as big as even now men have the power to vouch;
just so do all these things through one another race,
and, roaming, visit other places constantly;
we do not reach the middle place in union
But whensoever Hatred to the vortex' utmost depths
arrives, and Love arises in the whirlwind's midst,
in Her then all these things unite to be just one.
Strive so my words reach not your ears alone, E
and as you hear from me true facts take note: Pm1
I'll put before your eyes how She augments
with larger form life's union and increase,
and all that still remains of this creation,
first in the wild tribes of beasts that roam the hills,
then in the double race of man, then in the fruits
of rooted plants and grapes that mount the vine.
From these take in your mind proofs truthful of my words. E
You'll see life's union and increase, Pm2
because of Hate, wreak deeds of separation. D
This first the mass of mortal limbs makes clear;
at one time we unite in Love with all
the limbs that bodies have when life is blossoming;
another time again, split up by evil Strife,
they wander each apart amid life's breakers.
Just so it is for plants, and water-dwelling fish,
and beasts that sleep on hills, and fowls that mount on wings.
. . . . . .

Origin of life:

T3 ends by exploring the origin of evolving lineages at planetary level. First, it defines a timeframe for dynamic unification ('many ages previous must elapse') and then uses a simile to describe how biological wholes change (the sun, moon and stars changing place in a cycle). Second, it focuses on 'untrodden' Earth, a time when life was emerging and there were no lineages that could spring from an organismal ancestor (even LUCA). Third, it is here where T3 again (crucially) introduces the network paradigm, but now describing it in dynamic fluid manner ('things' racing through one another, roaming, and visiting other places). In absence of lineages and modern organisms, networks must now depict molecular modules being recruited to form complex cellular machinery, as evolutionary genomics, computational biology, systems science, and astrobiology are now suggesting. Thus, networks describe the origin of molecular parts of emerging cellular wholes (metabolism, molecular functions, proteins, nucleic acids), again with the same double tale of unification and diversification responsible for levels of biological organization. The word 'union' appears in the translation three times after its initial debut describing natural selection (line 236). At the end of T3, 'we' (the living world) fail to reach the middle place in 'union' as the molecular world of diversified parts unfolds, but then the unifying force ('Love') in constant flux 'unite things to be just one' into an entity (LUCA) that could then diversify.

Accretion:

The first promise (Pm1) presents the expectation that the dynamic force of molecular 'union' introduced in T3 (which applies to the origins of life) will 'augment with larger form life's union and increase' and that it will augment 'all that still remains of this creation'. Pm1 then represents a statement of molecular growth (accretion), in which molecular parts unify to form more complex molecular structure (e.g. proteins, ribosomes, membranes, cells). Interestingly, this process of accretion translates into a struggle intimated by 'all that still remains', which is in line with the 'natural selection' theme introduced at the beginning of the poem. In Pm1, the growth process that survives selection is illustrated with organisms (tribes of beasts, double race of man) and parts of organisms (fruits of rooted plants, grapes that mount the vine), making it clear that growth is a universal generality that manifests both in the presence or absence of organisms.

Modules:

The second promise (Pm2) presents the expectation that the unifying force responsible for 'life's union and increase' will be counteracted by the diversification force ('Hate') of the double tale, which will generate new parts (modules) for new generative cycles or unification and diversification. This dynamic double tale of parts and wholes materializes into a metaphor of a 'mass' of mortal limbs, referred to as 'illustrious bulk' that 'wanders separately in the surf of life' in other translations. We argued this illustrious 'mass' refers to the triskelion motif of rotational symmetry used as symbolic device in Empedocles' homeland at his time, which now decorates the flags of Sicily and the Isle of Man (see appendix in Caetano-Anollés and Janko, 2021). The spiral dynamics of limbs in Pm2 describes how parts 'wander' by recruitment in wholes (exemplified by plants, fish, beasts, fowls), an interpretation that is consistent with the extensive role that horizontal processes of recruitment and horizontal exchange have in organismal evolution.

. . . . that at life's end the limbs of all
fall from each other apart and meet their fate,
much though we will it not, from grim necessity,
as we decay. Though beauteous Love may hold us now,
the harpies with death's lots will come for us.
Woe that some ruthless day did not destroy me first,
before I used my claws to wreak dread deeds for food!
Now to no end my cheeks I wet with tears.
For we shall reach the unplumbed gyre, I fear,
and though men wish it not they'll have uncounted pains.
But we'll embark once more upon our tale F
When once the tireless flame did chance upon
all things, and caused their painful intermixture,
then creatures too progenitive were born
in every way, whose remnants still the dawn beholds.
When aither mixed did reach the utmost edge,
then birds flew up with shrieks and cries
tumultuous, but beasts, whose lot is lairs in earth
and grass, were born where earth was swathed around.
As when . . . . ,
a smith . . . .
. . . .
Just so the elements appear in different beasts
First in the whelks with heavy backs that graze the sea
and in their stony mantles, then in oysters –
there you will see earth dwelling on the top of flesh.
Again, the armour-plate of strong-backed sea-crabs,
also the stone-skinned conches' and the tortoises' shells,
and spears of hornèd deer that roam the hills.
But listing all such creatures I'd not end.
from which . . . .,
and this . . . .,
. . . .

Life cycles

The end of Pm2 uses decaying limbs as analogy to the problem of life history, the series of changes undergone by organisms during their lifetime. Here, the human life cycle is personified with the problem of death amid the continuity of life ('steady cycle', line 244). The poetic rendition is used to highlight the struggle for existence of the 'Natural selection' theme introduced at the beginning the poem.

Fossils:

The fulfillment of the promises (F) put forth in Pm1 and Pm2 embody finding evidence in remnants left behind by 'progenitive' creatures that existed in the past. Therefore, F requires identifying footprints of organismal history, either fossilized remnants in Earth's crust (e.g. microfossils, fossilized bones, geochemical, biochemical and biomarker data), living fossils (e.g. parts of biological macromolecules, such as sequences or structures of proteins, that are evolutionarily conserved), or evolutionarily conserved phenotypes in molecules and organisms (traits that inform about the past). Remarkably, the assembly of the last few papyrus fragments at the end of F recounts examples of characteristic Earth-like structures (phenotypes, not organisms) arising from the double tale as 'earth dwelling on the top of flesh' (line 356). One commonality of all mentioned organismal structures is filamentous keratin, a protein macromolecule that is part of a broad family of fibrous structural proteins. Remarkably, Empedocles' chosen examples involve a molecular trait that is subject to complex vertical and horizontal evolutionary forces, which make keratins unfold along the evolutionary paths of a network. We contend modern science has fulfilled the promises put forth in Empedocles' ancient papyrus.

A note on thematic order: The order of indexed themes is tantalizing. The main thesis at the beginning of the poem puts forth a double tale capable of explaining unification and diversification processes responsible for the generation of levels of biological organization. These processes are evolutionary and are constrained by natural selection. The second and third theses of the poem illustrate the double tale with evolutionary lineages, which unfold hierarchy and reticulation in a network of life (*tela vitae*). Finally, the poem recounts the history of the living world going back to a time prior to the last universal common ancestor (LUCA) and illustrates how the double tale explains the rise of primordial complexity at the dawn of life. The two promises that follow describe expectations of the theory, a process responsible for molecular and organismal growth and a process of innovation and diversification. The poem ends with fulfillment of promises in fossilized remnants left behind, including living fossils in the form of evolutionarily conserved phenotypes.
